# Multiple recent co-options of Optix associated with novel traits in adaptive butterfly wing radiations

**DOI:** 10.1186/2041-9139-5-7

**Published:** 2014-02-05

**Authors:** Arnaud Martin, Kyle J McCulloch, Nipam H Patel, Adriana D Briscoe, Lawrence E Gilbert, Robert D Reed

**Affiliations:** 1 Department of Ecology and Evolutionary Biology, Cornell University, Ithaca, NY 14853, USA; 2 Department of Ecology and Evolutionary Biology, University of California, Irvine, CA 92697-2525, USA; 3 Department of Molecular and Cell Biology, University of California, Berkeley, CA 94720-3140, USA; 4 Department of Integrative Biology, University of Texas, Austin, TX 78712, USA

**Keywords:** *Heliconius*, Adaptive radiation, Evolutionary novelty, Pattern evolution, Co-option, Sexual dimorphism

## Abstract

**Background:**

While the ecological factors that drive phenotypic radiations are often well understood, less is known about the generative mechanisms that cause the emergence and subsequent diversification of novel features. *Heliconius* butterflies display an extraordinary diversity of wing patterns due in part to mimicry and sexual selection. Identifying the genetic drivers of this crucible of evolution is now within reach, as it was recently shown that cis-regulatory variation of the *optix* transcription factor explains red pattern differences in the adaptive radiations of the *Heliconius melpomene* and *Heliconius erato* species groups.

**Results:**

Here, we compare the developmental expression of the Optix protein across a large phylogenetic sample of butterflies and infer that its color patterning role originated at the base of the neotropical passion-vine butterfly clade (Lepidoptera, Nymphalidae, Tribe: Heliconiini), shortly predating multiple Optix-driven wing pattern radiations in the speciose *Heliconius* and *Eueides* genera. We also characterize novel Optix and Doublesex expression in the male-specific pheromone wing scales of the basal heliconiines *Dryas* and *Agraulis*, thus illustrating that within the Heliconinii lineage, Optix has been evolutionarily redeployed in multiple contexts in association with diverse wing features.

**Conclusions:**

Our findings reveal that the repeated co-option of Optix into various aspects of wing scale specification was associated with multiple evolutionary novelties over a relatively short evolutionary time scale. In particular, the recruitment of Optix expression in colored scale cell precursors was a necessary condition to the explosive diversification of passion-vine butterfly wing patterns. The novel deployment of a gene followed by spatial modulation of its expression in a given cell type could be a common mode of developmental innovation for triggering phenotypic radiations.

## Background

Adaptive or phenotypic radiations are defined by ‘the evolution of phenotypic diversity within a rapidly multiplying lineage’ [1,2] and form crucibles of natural variation for the study of distinctness, as illustrated by Darwin’s work on Galapagos finches [3]. Ecological arguments involving external (biotic or abiotic) factors are traditionally invoked to explain why phenotypic disparity is maintained over time [2], but they fail to explain the variation-generating mechanisms that allow these rapid bouts of evolution to occur. Recently, a handful of studies focusing on the *intrinsic causes* of adaptive radiations showed that diversification could follow relatively simple developmental innovations [4-9]. What principles underlie the emergence of new features, and how do these features further diversify [10,11]? A meaningful answer to this question may come from comparisons between many organisms, and adaptive radiations assist us in this task by providing a replicated and diverse template of phenotypic variation [12]. *Heliconius* butterflies are perhaps one of the best studied examples of a phenotypic radiation [13-15]. The genus comprises 40 species distributed across the Neotropics and is characterized by a variegated mosaic of wing patterns that vary both among and within species [13,16]. Wing patterns commonly function in nature as aposematic traits (that is*,* as signals displayed to warn potential predators of danger), and butterflies such as *Heliconius erato* and *Heliconius melpomene* are a well-known example of this phenomenon [17]. Both species signal their unpalatability by displaying similar wing patterns that are indistinguishable from each other, are endemic to a restricted geographical range, and almost always include red coloration [18]. This Müllerian (that is*,* reciprocal) mimicry provides a strong adaptive advantage and predators learn to avoid all co-mimics [19]. In fact, most *Heliconius* species display extensive geographic variation in discrete wing patterns; more than 300 *Heliconius* regional morphs are recognized by their distinct wing pattern phenotypes and are each locally involved in complex mimicry rings with other co-mimetic butterflies [16,19,20].

Many ecological factors explain the diversity of *Heliconius* butterflies [21] such as their co-evolution with host plants of the *Passiflora* genus [22,23] and their participation in dynamic co-mimetic communities [19,24]. However, these extrinsic factors remain mute regarding the mechanisms that generate wing pattern variation in the first place. Mapping experiments taking advantage of intraspecific variability in the genus have begun to shed light on this question, and have revealed a monogenic (Mendelian) genetic architecture for discrete allelic variation in red patterns [25-27]. In particular, it was found that cis-regulatory evolution of the *optix* transcription factor is responsible for the presence/absence of red wing patterns in convergent forms of the *H. erato* and *H. melpomene* species, as well as in the divergent *Heliconius cydno and Heliconius pachinus* morphs [28,29]. Genetic variation at the *optix* locus explains a large fraction of the diversity of wing patterns in the genus, and was also found to contribute to the convergent evolution of mimetic forms by introgression of *optix* alleles in related lineages, resulting in a scattered phylogenetic distributions of adaptive patterns [15,18,30-32]. Thus, *optix* incarnates an emblematic example of ‘hotspot of evolution’ where the same gene has repeatedly caused phenotypic variation [28,33]. Interestingly, some of the variable red patterns controlled by *optix* are not only involved in predation avoidance, but are also drivers of reproductive isolation by assortative mating [34-36], suggesting that *optix* allelic variation may directly influence speciation rates.

Despite their ecological significance, little is known about the evolutionary origins of *optix*-positive red color patterns. Drawing conclusions from morphological analyses alone has been challenging because the wing pattern architecture of *Heliconius* is divergent relative to the nymphalid ground plan [27,37,38] - the system of wing pattern homologies that is otherwise broadly conserved across the family Nymphalidae [39,40]. Here, we generated a polyclonal antibody directed against the Optix protein and compared its immunoreactivity in the developing wings of *Heliconius* and other butterflies. We found that the role of Optix in color patterning is relatively recent, making it a synapomorphy of neotropical passion-vine butterflies (tribe: Heliconiini), and that Optix was also associated to the development of novel, sexually dimorphic scale structures in basal heliconiines. In the light of previous work, we conclude that co-option of the Optix transcription factor into the color pattern formation process was a key pre-requisite for the diversification of red wing patterns. The co-option of Optix is thus consistent with a two-step innovation/diversification model, where the evolution of a novel character followed by its spatial modulation generated a wide array of distinct wing patterns.

## Methods

### Polyclonal antibody production

The nucleotide sequence of the *H. erato optix* gene [GenBank: JN102349.1] was modified using codon degeneracy to artificially lower high GC content without affecting the native amino-acid sequence. The corresponding DNA fragment was generated by gene synthesis (Genewiz,South Plainfield, New Jersey, USA) and cloned into the pQE-30 expression vector (Qiagen, Valencia, California, USA). A 6xHis-Optix fusion protein was expressed in bacterial cultures, extracted in 8 M Urea and purified using Ni2+ affinity column. An insoluble precipitate was recovered upon dialysis and approximately 1 mg was injected in a rat for immunization (5 injections of 200 ug each across 69 days; Covance, Princeton, New Jersey, USA).

### Butterfly tissues

Butterfly pupae either originated from individuals collected in southern California, from laboratory colonies maintained at University of Texas - Austin, or from a butterfly farm in Costa Rica [see Additional file 1: Table S1]. Whenever possible, the time and day of pupation was monitored. Pupae were then kept at room temperature (23 to 26°C) and dissected on the fourth or fifth day after pupation, a stage at which wings are resistant to shearing while also being permeable to antibodies. Individuals originating from Costa Rica were shipped as untimed pupae and arrived in the laboratory after three days of transit. These pupae were approximately staged by screening their external eye morphology upon receipt. Pupae not presenting mature ommatidial structures (that is*,* those at an earlier stage of development) were immediately dissected. A portion of these then yielded pupal wing tissues at stages that were early enough for antibody permeability [see Additional file 1: Table S1]. The immunohistochemical signal gradually decreases as pupal development proceeds to a point where scale cells begin to mature, the wing epithelia become an opaque white, and the forewings easily detach from the cuticle upon dissection. For all species, dissections performed before this stage resulted in reproducible immunodetection of Optix.

### Tissue preparation and immunohistochemistry

Pupae were cold-anesthetized and dissected in PBS. Pupal forewings were incubated in fixative (PBS, ethylene glycol 5 mM tetraacetic acid, 0.01% Triton X-100, 1.85% formaldehyde) for 30 min at room temperature while still attached to their cuticle. Similarly, and in order to maintain flat wing tissues, hindwings were kept attached to their pupal case and fixed for at least 5 min as such, detached by dissection, and then the remainder of the 30 min fixation was completed on free-floating wings. Fixed wings were washed five times with PBST (PBS, 0.5% Triton X-100) and stored at 4°C for up to two months. Wings were cleaned from remnants of peripodial membrane with fine forceps and incubated in blocking buffer for 2 hrs (PBS, 0.5% Triton X-100, 5% Normal Goat Serum; at room temperature), incubated overnight at 4°C with anti-Optix polyclonal rat serum (dilution 1:3000), washed six times with PBST, incubated for 2 hrs at room temperature with an anti-rat IgG Alexa555 (Cell Signaling Technology, Danvers, Massachusetts, USA; 1:2000 dilution), and washed extensively in PBST. Nuclear counterstaining was performed with DAPI, and the wings were mounted in glycerol (PBS, 60% glycerol, 2 mM ethylenediaminetetraacetic acid). F-actin counterstaining was performed using phalloidin conjugated to green fluorescent Oregon Green 488 dye (Life Technologies, Carlsbad, California, USA; 1:400 dilution). For immunostaining in eyes and optic lobes, whole heads were fixed in 4% paraformaldehyde, sucrose protected, and frozen for sectioning. Horizontal sections of 14-μm thickness were made using a cryostat and used for immunohistofluorescence using a previously described procedure [41]. A rabbit anti-UV opsin primary antibody [41] and an anti-rabbit IgG Alexa488 secondary antibody (Cell Signaling Technology, Danvers, Massachusetts, USA; 1:2000 dilution) were used as a positive control in adult eyes. The anti-Doublesex monoclonal mouse antibody [42] was a kind gift of C. Robinett (Janelia Farms Research Campus), was used at a 1:200 dilution and was detected using an anti-mouse IgG Alexa 488 secondary antibody (Cell Signaling Technology, Danvers, Massachusetts, USA; 1:2000 dilution).

### Microscopy

Stained tissues were imaged with a Zeiss Axioplan 2 epifluorescence microscope and with a Zeiss LSM 700 confocal microscope. Tiled images were assembled using either the Photomerge function of Adobe Photoshop CS3 (epifluorescence imaging), or were acquired using the Tiled Scan function in the Zeiss ZEN software (confocal imaging). Images were submitted to a linear contrast adjustment to eliminate background illumination.

Other methods are described in the Additional file 1: Methods section of this manuscript.

## Results

### Specific detection of the Optix protein during wing development

**Figure 1:**
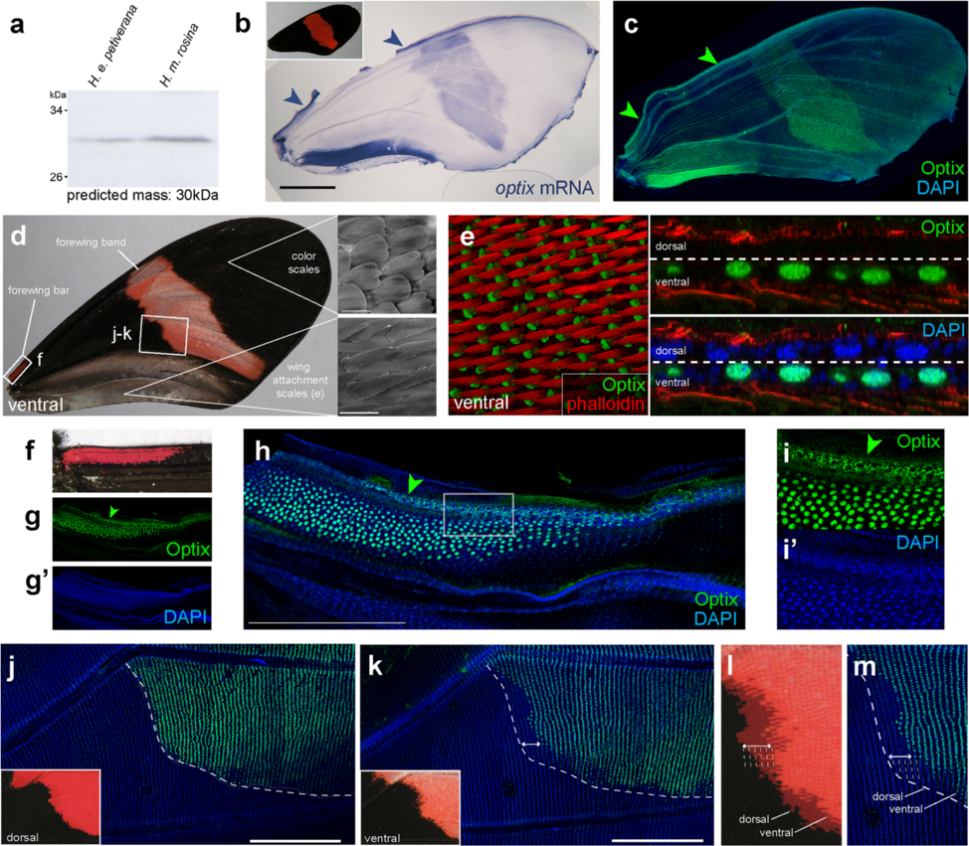
**Immunodetection of the Optix protein in *Heliconius erato *forewings. (a)** Western blot on protein extractions from *H. erato petiverana* and *H. melpomene rosina* forewing using the anti-Optix serum. The predicted size of the *Heliconius spp*. Optix protein is 30 kDa. **(b)** Detection of *optix* by mRNA *in situ* hybridization in a *H. erato petiverana* forewing. **(c)** Immunofluorescence using the anti-Optix serum reproduces the mRNA pattern. Blue DAPI is a nuclear counterstain. Arrowheads **(b-c)**: forewing blade expression. **(d)** Organization of a ventral *H. erato petiverana* forewing featuring two red patterns (forewing band and forewing bar) and the posterior field of wing coupling, spear-shaped scales. **(e)** Sectional visualization of Optix expression in the wing coupling scale cell precursors. **(f, g)** Magnified view of the adult forewing bar, as a small ventral pattern situated at the base of the wing costa. **(h, i)** Optix detection in the corresponding region marks the precursor scale cells of the presumptive red forewing bar. Arrowheads: Optix-positive, small interstitial cells of the wing blade. **(j-m)** Side-specific visualization of Optix expression on two different confocal plans exposes dorso-ventral offsets in forewing bar boundaries. The dotted line highlights the relative position of the dorsal pattern boundary on ventral side focal plans. In panel l, ventral and mirror-imaged dorsal views are super-imposed using vein landmarks. Scale bars: d, 20 μm; h, j, k, 500 μm.

A polyclonal antibody serum was generated in a rat immunized with the *Heliconius* Optix protein. The crude serum immunodetected a band of the expected size (30 kDa) in a western blot (Figure 1a), and immunofluorescence in the developing wings of *H. erato petiverana* precisely replicated the *optix* mRNA expression pattern (Figure 1b-c). No signal was observed in *Drosophila melanogaster* and *Danio rerio* embryos (data not shown), suggesting that the polyclonal serum targets epitopes that are Lepidoptera-specific and is also unlikely to detect other lepidopteran SIX family transcription factors. Wing veins and scale sockets produced signals in several negative controls [see Additional file 1: Figure S1], and are thus non-specific signals inherent to our pupal stage immunodetection assays. The scale socket artifactual staining is stage-dependent and does not interfere with the specific detection of Optix at more internal focal planes using confocal imaging, or during whole-wing imaging at lower magnifications using epifluorescence imaging.

Optix was detected during pupal wing development in discrete expression domains that were not associated with color scales. As previously noted [29], Optix marks the large nuclei of cells that differentiate into spear-shaped acute scales (Figure 1d-e) thought to be involved in wing coupling by frictional effect [43]. Optix was also detected in an anterior domain of small interstitial cells that run along the wing blade of the forewing (Figure 1f-i). In terms of color patterns, Optix was associated with presumptive red scales, including in a small ventral red bar at the base of the forewing costa (Figure 1f-h). We confirmed presumptive color scale correspondence with cellular resolution and in a dorsal-ventral specific-manner. For instance, the red median forewing band of *H. erato petiverana* differs on its proximal boundary by up to six scale rows between the ventral and dorsal sides. This difference is reflected by corresponding shifts of Optix immunolocalization (Figure 1j-m). These observations provide further verification at the protein level that *optix* is a selector gene switching on red scale color phenotypes [29,44].

### Conserved color pattern-independent roles of Optix across nymphalid butterflies

**Figure 2:**
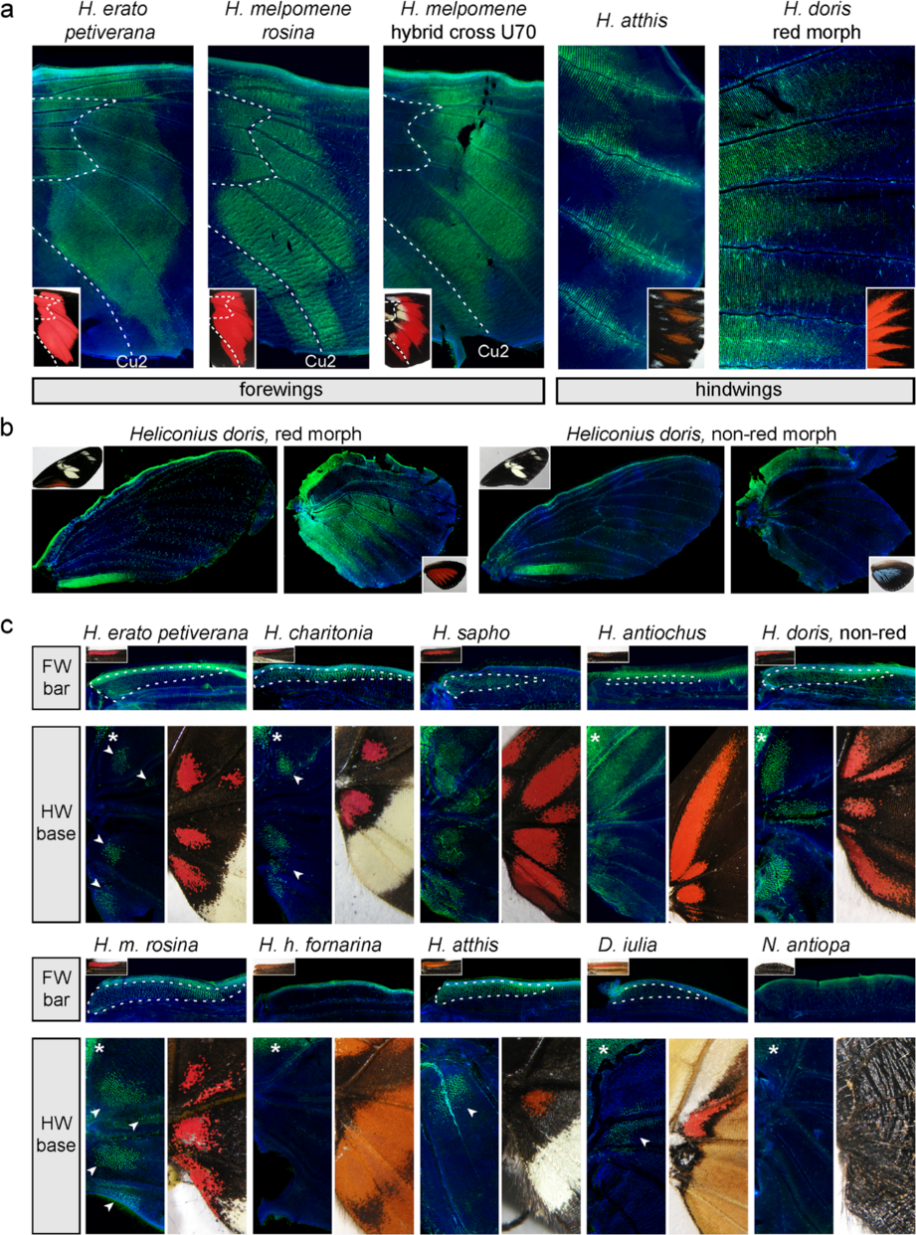
**Optix reveals developmental homologies between wing patterns. (a)** Optix expression is tightly associated with various *Heliconius* red wing patterns. Notice the interspecific difference in pattern boundaries relative to the Cu2 vein (dotted lines). The hybrid cross U70 is described in Figure 3. **(b)** Optix expression in the polymorphic *Heliconius doris* butterflies. Insets feature dorsal views of adult patterns. **(c)** Conserved association of Optix expression with red ‘proximal complex’ patterns (forewing bar and hindwing basal spots) across Heliconiini. Asterisks: color pattern-independent expression of Optix in dorsal patches of presumptive acute scales. Insets feature ventral views of adult patterns.

We compared the expression of Optix during the pupal wing development of 13 butterfly morphs and species of the family Nymphalidae (Figure 2; [see Additional file 1: Figure S2]). In each case, Optix marked the presumptive wing coupling scales on ventral forewings and dorsal hindwings. The shape and size of these fields of specialized scales are sexually dimorphic in *Heliconius*, with a larger size observed in males compared to females [see Additional file 1: Figure S3; Reference 43]. Corresponding variability in the size and relative positions of Optix positive cell fields were observed; for instance, immunodetection of Optix in *H. hecale fornarina* and a *H. melpomene* hybrid [see Additional file 1: Figure S2] revealed domains of expression extending anteriorly on the forewing and posteriorly on the hindwing that are characteristic of male wing coupling patches [see Additional file 1: Figure S3]. In addition, the Optix antigenicity of the forewing wing blade interstitial cells was observed in all tested species. Expression of Optix in the wing blade and wing attachment scale fields replicate previous *optix* mRNA localization results [29], uncover broadly conserved features of butterfly wing pattern development, and provide internal controls for the successful detection of Optix in wing tissues.

### Optix reveals homology of red wing patterns among passion-vine butterflies

We replicated the previous observation that Optix expression is associated with the development of red scale patches in *Heliconius* (Figure 2a), and is in large part responsible for convergent evolution of the Central American *H. erato petiverana* and *H. melpomene rosina* co-mimics [29,45]. To further assess wing pattern homologies across the genus *Heliconius* we examined the association of Optix with red coloration to additional species and morphs (Figure 2; [see Additional file 1: Figure S2]), including *Heliconius atthis*, which has remarkably divergent red patterns on the hindwing. One notable species we examined, *Heliconius doris*, is characterized by polymorphic populations where ventral hindwings either display a conspicuous red ray pattern or a more narrow blue or green pattern - discrete variation that is observed within single broods [46]. This variability was present in the stock we assessed (78% red versus 22% non-red individuals; N = 33 emerged butterflies), and was paralleled by Optix immunodetection in a large and rayed ventral patch in 70% (N = 10) of assessed individuals (Figure 2b). Preliminary observations suggest a Mendelian mode of inheritance of *H. doris* wing patterns [46], and our result suggests that allelic variation in *optix* or its upstream regulators is a candidate mechanism for this case of morphological polymorphism.

Optix was consistently detected (with one exception, see below for discussion of *Heliconius hecale fornarina*) in what we term the ‘proximal complex’, consisting of the red costal forewing bars and of basal hindwing red spots, both situated on ventral wing surfaces (Figures 1j-m and 2c). It is unclear whether such small color elements always have an adaptive function in mimicry, but the clear homology between this set of Optix-positive ventral patterns highlights a conserved theme of an otherwise multifarious array of *Heliconius* wing patterns. This character has deeper roots in the phylogeny, as we positively identified a homologue of the proximal complex in *Dryas iulia*, which shows Optix-positive scale precursors that mark ventral patches of salmon pink scales. The basal position of *D. iulia* in the Heliconiini clade [see Additional file 1: Figure S4] implies that a color patterning role of Optix preceded the *Heliconius* radiations, consistent with the observation that many species of the Heliconiini clade harbor unambiguous positional homologs of the proximal complex [see Additional file 1: Figure S5]. Similar patterns are not found in heliconiine outgroup lineages, suggesting that the Optix-positive proximal complex is a synapomorphy of the Heliconiini clade of passion-vine butterflies [see Additional file 1: Figure S5].

### Optix is spatially regulated by the *N* locus

**Figure 3:**
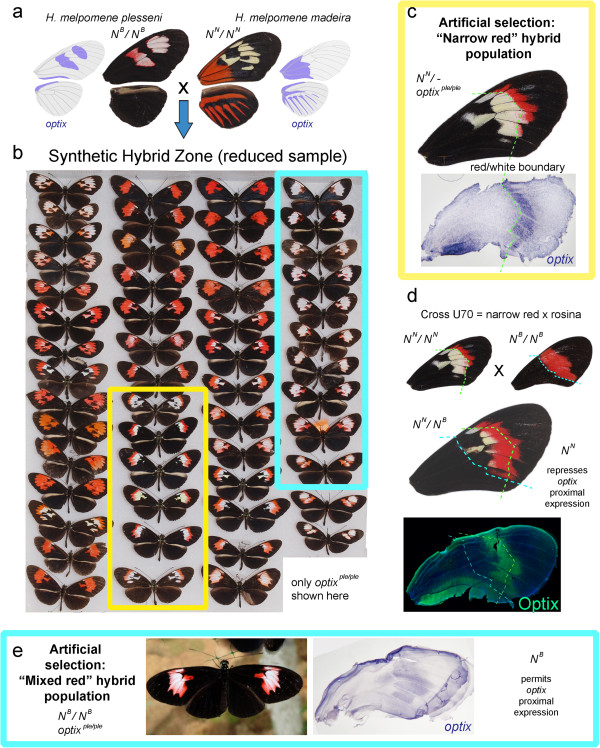
**Epistatic regulation of *optix *in hybrid patterns. (a-b)** A synthetic hybrid zone was created by interbreeding *Heliconius melpomene plesseni* and *H. melpomene madeira* butterflies in a large greenhouse. Expression of *optix* in founders is reproduced from *in situ* hybridization [29] and the *plesseni* white scales expresses *optix*. The *N* locus that modulates the color composition of the median forewing: non-Amazonian (non-rayed) races such as *plesseni* bear *N*^*B*^ alleles [48], while Amazonian (rayed) races such as *madeira* bear *N*^*N*^ alleles that result in a distal localization of the forewing red band [14,48]. Panel b shows a sample of the phenotypic diversity observed on forewings of this hybrid population. Only butterflies that were homozygous for the *plesseni optix* allele are shown here (dominant rayed alleles excluded). Hindwings show no pattern variation in this population and are not shown. **(c)** Artificial selection of butterflies showing a band of distal red (yellow box) generated a stable population called ‘Narrow red’. The distal localization of red is explained by *N*^*N*^ alleles that repress proximal expression of *optix*. **(d)** Crossing a ‘narrow red’ individual with a *N*^*B*^/*N*^*B*^ butterfly does not recover the full expression of the forewing band red in the F1 progeny, in which Optix expression is limited to the distal portion of the white/red boundary. The absence of fully red forewing bands similar to the parental *rosina* pattern in the F1 shows the ‘narrow red’ parent is *N*^*N*^-homozygous. **(e)** Artificial selection of butterflies showing a mixture of red and white scales in the forewing band (blue box) generated a stable population called ‘Mixed red’. In the presence of *N*^*B*^ alleles derived from *plesseni*, proximal *optix* expression (for instance overlapping with the discal cell) and only delimited by the total shape of the forewing bar.

In *H. melpomene* an epistatic interaction between *optix* and the modifier locus *N* results in a narrow forewing red band [14,47]. This two-locus system is interesting as it can generate novel patterns by hybridization. For instance, introgression of a *H. melpomene optix* allele into the *cydno* clade generated a chimeric phenotype due to epistasis with *N* in the hybrid species *Heliconius heurippa*, resulting in a distal localization of forewing red [47]. We sought to determine if this activity of *N* is due to an effect on the distribution of the *optix* gene products, or simply to the repression of red pigment synthesis in an otherwise *optix*-positive pattern, as seen in *H. melpomene plesseni*[29]. To distinguish between these two scenarios we used an artificial hybrid zone strategy [31] to generate a composite phenotype analogous to the yellow/red forewing pattern of *H. heurippa* (Figure 3). We found that a genetic factor of Amazonian origin analogous to the *N*^*N*^ allele [14,48] repressed a proximal domain of *optix* expression, resulting in a ‘novel’ white/red pattern. We thus conclude that *N*, which has not yet been characterized at a molecular level, is a direct or indirect regulator of *optix* expression.

### Optix pinpoints regulatory differences between mimetic orange and red patterns

The hindwings of the butterfly *H. hecale fornarina* show a claw-shaped ventral pattern that is reminiscent of a similar pattern observed in *Heliconius cydno galanthus*[49]. Both butterflies co-occur in a large distribution range that spans southern Mexico, Guatemala, and El Salvador, but differ by the yellow versus white color of their forewing bands. However, spectrometer reflectance measurements performed on the *H. cydno galanthus* and *H. hecale fornarina* red hindwing patterns [see Additional file 1: Figure S6] are almost identical and suggest that natural predators are unable to discriminate between these signals. We previously found that the orange-brown claw pattern of *H. cydno* expresses *optix*[29], and thus wanted to determine whether the *H. hecale fornarina* pattern would reveal a similar expression pattern. Unexpectedly, we found that Optix was neither expressed in the hindwing claw-shaped pattern nor in the short costal forewing bar (Figure 2c). This negative result indicates that *H. hecale fornarina* orange/brown color patterning may follow a divergent process. For example, Optix expression could occur at later developmental stages that could not be assayed by our immunostainings. Whatever the explanation, the color patterning role of Optix in silvaniforms - the tiger-like butterflies similar to *H. hecale*[49] - remains to be determined.

### Optix marks presumptive male-specific wing scales in basal Heliconiini

**Figure 4:**
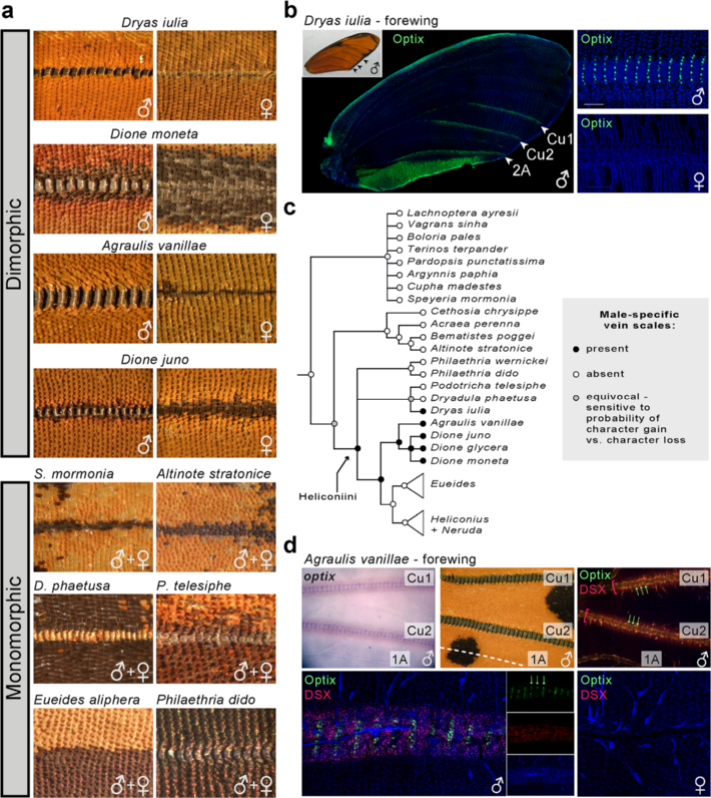
**Sexual dimorphism in Optix-positive male vein structures among basal Heliconiini. (a)** Examples of dorsal forewing Cu1 veins among Heliconiinae. Veins of the *Dryas*, *Agraulis* and *Dione* genera show striated pheromone-spreading structures in males only, as previously described in *Agraulis*[50,51]. **(b)** Sexual dimorphism in Optix expression associated with the male-specific striated vein structures of *Dryas iulia* (see also panel **a**). Scale bars: 100 μm. **(c)** Character mapping and ancestral reconstruction of vein sexual dimorphism in Heliconiinae with probability of loss ≥10x probability of gain (black: presence; white: absence; grey ancestral nodes: equivocal). (**d**, upper panels) Expression of Optix and Doublesex in *Agraulis vanillae* forewings. *optix* mRNA (purple staining) and protein products (green arrows) marks male-specific scale cell precursors during pupal wing development. Vein epithelia of dimorphic veins express Doublesex (red brackets). Male forewing *1A* veins are devoid of adult striated organs and lack Optix and Doublesex expression. (**d**, lower panels) Sexual dimorphism in Optix and Doublesex in male forewing veins.

Immunodetection of Optix in *Dryas* marked rows of 6 to 8 scale precursor cells that are dispersed along the Cu1, Cu2, and 2A forewing veins (Figure 4a-b). This expression pattern was observed in male but not in female individuals (N = 5; 3 males, 2 females), suggesting a case of sexual dimorphism. Examination of *Dryas* adult wings revealed the presence of male-specific, striated features on the M3, Cu1, and 2A veins; these features resemble pheromone-spreading structures that were previously described in *Agraulis vanillae*[50,51], another passion-vine butterfly. Upon closer examination, we noticed that Optix was expressed in macronuclear cells (characteristic of scale cell precursors) that cover the dorsal side of the veins, forming rows of 6 to 8 cells that align with extravenous Optix-negative scale cell precursors. This relative arrangement was compared to dissections of adult male veins and indicates that Optix marks the scale precursors of the male-specific ‘scale section-1’ [50]. These cells give rise to single rows of 6 to 8 densely packed scales of gold and black color that act as a protective sheath for fragile brush-bearing scales situated underneath [50,51]. This combined scale arrangement is coupled with a pore system and is likely involved in the dissemination of male pheromones; it is also possible that the scale section-1 prevents excessive evaporation of the secreted volatiles [50]. Striated expression of *optix* in this scale system was replicated in *Agraulis*, and we observed similar male-specific structures in the *Dione* genus (Figure 4b). Current tree topology of basal Heliconiini suggests that these Optix-positive, sexually dimorphic organs are ancestral in Heliconiini and were lost at least three times during the subsequent radiation of this lineage (Figure 4c).

To gain further insights into the development of sexual dimorphism in the wing veins of these butterflies, we sought to test the expression of Doublesex, a transcription factor that has been proposed as a master regulator of sex-specific gene expression and development [52]. An antibody targeting the *Drosophila* Doublesex DNA-binding domain was recently developed using a conserved antigen [42]. We hypothesized that this antibody would be likely to cross-react with a butterfly Doublesex orthologue, as a BLASTP analysis using the antigenic peptide sequence [42] as a query resulted a single hit with 52 out of 59 conserved residues (88% conservation) against the *Heliconius melpomene* annotated protein set [15]. Remarkably, immunodetection of Doublesex in *Agraulis* developing pupal wings specifically marked the dorsal epithelium of the dimorphic veins that include the Optix-positive scale cell precursors (Figure 4c). Only male individuals showed a positive signal among assessed individuals (N = 6, 3 males, 3 females), and all the male veins that are devoid of striated structures - that is*,* the forewing 1A and all the hindwing veins [50,51] - showed no Doublesex signal, revealing a tight association of Doublesex expression with sexually dimorphic structures in this system. This result suggests that Doublesex activates the development of the male-specific wing scales via its sex-limited expression, and we speculate that Doublesex could be a transcriptional co-regulator of *optix* in basal heliconiines.

### Optix is expressed in neural progenitors of the optic lobe

**Figure 5:**
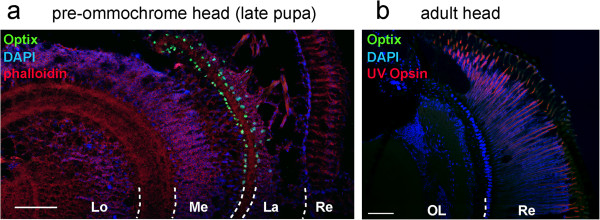
**Optix is transiently expressed in a small population of optic lobe neurons. (a)** Expression of Optix in *Heliconius* optic lobes at the pre-ommochrome stage. Similar results were obtained for *H. doris* and *H. sapho*. **(b)** Lack of Optix expression in optic lobes and retina at the adult stage. Immunodetection of the photoreceptors UVRh1/2 is shown here in ommatidia as a positive control. This negative result was replicated in *H. melpomene*, *H. cydno*, and *H. doris*. La, lamina; Lo, lobula ; Me, medulla; Re, retina. OL, optic lobe. Scale bars: 100 μm.

It was previously proposed that butterfly wing pigments originate from the redeployment in wings of biosynthetic modules already present in eyes [53]. Indeed, insect eyes express ommochrome pigments that also compose the yellow, orange, and red pigments of *Heliconius* wings [54,55]. Since *optix* is sufficient to induce ectopic eyes that include red pigmentation in flies [56], it was proposed that the co-option of a simplified *optix* eye regulatory module could have taken place in wings, therefore explaining the regulation of red ommochrome pigmentation by *optix* in *Heliconius*[57]. We assessed a variant of this ‘eye-to-wing’ co-option hypothesis by looking at optix expression in pupal eyes at the onset of ommochrome pigment deposition, as well as in adult retinae, where red pigment granules occur [58]. While *optix* is transcribed in wings at the pupal stage we examined [29,59], we did not detect Optix immunoreactivity in pupal or adult retinas of various *Heliconius* species (Figure 5). Interestingly, however, Optix was expressed at the pre-ommochrome stage in two single layers of neural progenitors that bordered the inner rim of the lamina and the outer rim of the medulla (Figure 5a). These neuropils are the first integrators of visual information, and it will be interesting to further use Optix as a marker of butterfly neuronal patterning and determine its role in visual processing.

## Discussion

The evolutionary origin of red wing patterns in *Heliconius* is unclear, as these elements seem divergent from the common pattern scheme shared by other butterflies [37]. Here, we clarify this issue by (*a*) arguing that Optix-positive patterns are a relatively recent novelty that seemingly appeared by co-option of Optix expression in color scale cell precursors, and (*b*) disentangling different genetic modes of pattern diversification that followed the original co-option event.

### Optix color patterns are an evolutionary novelty of passion-vine butterflies

**Figure 6:**
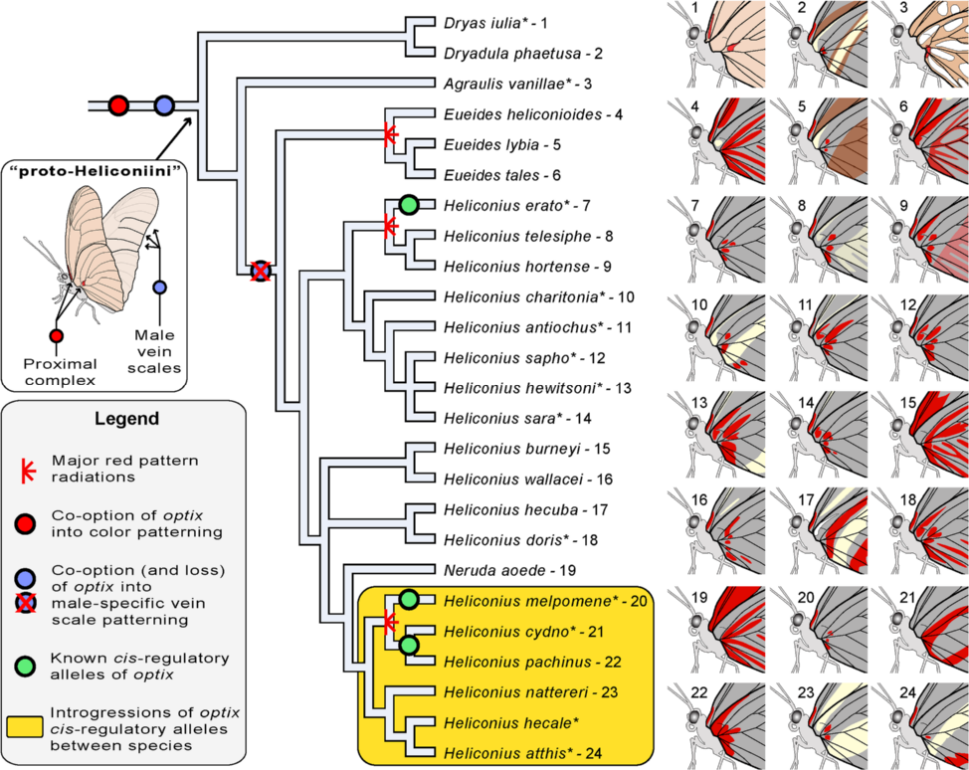
**Co-option followed by diversification of Optix-positive patterns.** Optix co-option into color scale cell precursors occurred prior to the Heliconiini radiation, notably resulting in ancestral patterns of the proximal complex (white box). These novel patterns subsequently diversified by cis-regulatory evolution of *optix* expression (as shown in branches marked by green bullets) and by changes at epistatic loci (see Discussion), explaining repeated diversifications of red patterns in the *Eueides* and *Heliconius* lineages. Finally, introgressions of *optix* alleles fostered the distribution of novel alleles between species, as shown in the melpomene-cydno-silvaniform clade (yellow box). The phylogenetic sampling and the associated insets highlight the diversification of ventral Optix-positive patterns, but similar principles apply to the dorsal side. Asterisks: species with known Optix expression. Current phylogeny [67] places Optix-positive vein scales observed in male *Dryas* and *Agraulis* as an ancestral, sexually dimorphic feature of Heliconiini, suggesting a co-option event followed by a secondary loss in the *Euiedes*-*Heliconius* lineage.

The co-option of a gene into a new context constitutes an unambiguous example of evolutionary novelty at the molecular level [60]. In particular, the re-deployment of developmental genes has been proposed to underlie the origin of novel traits such as butterfly wing patterns [10,61]. We observed Optix in the precursors of three types of scales: ancestral spear-shaped wing coupling scales [29]; specialized, male-specific, gold and black scales that are proposed to protect pheromone-spreading structures in basal Heliconiini [50]; and finally, the colored scales associated with pink (*Dryas iulia*) or red (*Heliconius spp*.) pigmentation across Heliconiini. We propose that each of these three Optix-associated scale phenotypes forms a distinct evolutionary character due to the divergent scale morphologies they underlie. Importantly, the recruitment of Optix expression into colored scale cell precursors of the ‘proximal complex’ is a synapomorphy of neotropical passion-vine butterflies (Heliconiini) and was detected in the basal genus *Dryas*, thus marking an innovation event associated with the Heliconiini radiation (Figure 6). In *H. erato* and *H. melpomene*, *optix* expression may activate the *cinnabar* and *ebony* pigment synthesis genes, as their transcripts show a strong spatial correlation with Optix-positive red pigmentation [54,55,59,62]. It will be interesting to further confirm the regulatory targets of Optix and to determine how many steps were necessary to achieve its color patterning role. As we were unable to validate the hypothesis of an eye-to-wing co-option event re-wiring an ancient pigmentation module [57], the mechanism underlying the recruitment of Optix into a color determination module remains to be elucidated.

In parallel with its recently evolved color patterning role, Optix also acquired a novel role in the specification of male-specific striated scale structures on the forewing veins of *Agraulis* butterflies as well as in the basal genus *Dryas*, suggesting a co-option event prior to the Heliconiini radiation (Figure 6). This implies that Optix has been repeatedly recruited in the specification of derived scale identities, highlighting a possible propensity of the *optix* gene to be recruited during the evolution of specialized scale types.

### Four modes of red pattern diversification in *Heliconius*

Optix expression implies that all the red patterns assessed in the *Heliconius* genus are variations of a conserved core process linking Optix expression in scale cell precursors to a narrow range of scale phenotypes (Figure 6). Under the conceptual framework of developmental homology [63] Optix scale cell precursor expression provides a character-defining mechanism (that is, the presence of red scales) that pinpoints an evolutionary novelty, whereas modulation of Optix spatial localization underlies the ‘character states’ of this trait and is a secondary effect of diversification. We summarize here our knowledge of the different modes of evolution of red patterns with a focus on *optix* regulation:

1. cis-regulatory evolution of *optix* has played a predominant role in the tinkering of novel red patterns. At least eight *optix* alleles with distinct pattern-switching effects have been identified by genetic mapping: four alleles in the *H. erato*/*H. himera* clade [27], two alleles in *H. melpomene*[25], and two alleles in the *H. cydno*/*Heliconius pachinus* sister species [26]. It is proposed that this allelic diversity results from the cumulative effect of many tightly linked *cis*-regulatory sequence variations organized in complex haplotypes and possibly compartmentalized into several pattern-specific *cis*-regulatory modules [45]. Following the observation that the genetic basis of phenotypic variation is highly repeatable [33], and that *optix* regulation maps to a large adjacent intergenic region that could reflect a potential for complex *cis*-regulatory structure, we extrapolate that direct *cis*-regulatory evolution of *optix* may extend to many cases of red pattern heterotopy (spatial shifts) in *Heliconius*.

2. While the *optix* locus tends to explain presence/absence polymorphism of a given pattern element (*for example,* the forewing band), pattern shape variation can sometimes be explained by epistatic effects with unlinked loci. Several examples have been characterized at various levels. For instance, the early expression of *WntA* during larval development determines the shape of the red forewing band [27,64]. We also show here that *N* spatially regulates Optix pattern shapes in *H. melpomene*.

3. Adaptive *optix* alleles have been shown to introgress across geographic barriers and to create taxonomic mosaics of pattern diversity, where a given pattern of single origin is scattered across the lineage phylogeny [18]. More surprisingly, lateral transfer by hybridization of wing patterns can occur beyond the species boundary [31]. Directly quoting the original report by Gilbert (2003), *virtually all departures from the common pattern themes within the cydno clade* (*for example*, *H. heurippa*, *H. timareta*, *H. pachinus*, *H. cydno weymeri form gustavi*) *can be explained by introgression of toolbox elements from H. melpomene*. Optix appears to be a core component of this toolbox, and recent genetic data has since confirmed that cross-species introgressions of *optix* alleles drive multiple examples of mimetic convergence in the ‘melpomene-cydno-silvaniform’ clade [15,30,32,65]. Introgressions do not directly cause the evolution of novel patterning alleles, but propagate adaptive variants across gene pools, and hybrids can display effects of novelty due to epistatic effects between wing pattern loci, as seen in *H. heurippa*[47,65]. Introgressions may be a major but previously unsuspected cause of phenotypic disparity across the animal kingdom, and *Heliconius optix* alleles emerge as a model system of choice to study this phenomenon [66].

4. In addition to the effects of loci that act upstream of *optix* and affect its localization, modifier loci acting *downstream* of *optix* may modulate its color output. A likely example occurs in *H. melpomene plesseni*, where an unidentified mechanism converts *optix*-positive scales into a highly-UV reflective scale field of white appearance [29,48]. This epistatic factor of *plesseni* origin is visible in an artificial ‘Mixed white’ hybrid population, where an unbroken forewing patch of white and red scales expresses *optix* (Figure 3e).

In summary, 1) cis-regulatory evolution of *optix* and epistatic interactions with upstream trans-regulatory factors affect the localization and shape of red patterns; 2) introgressions of *optix* variants maximize the phylogenetic distribution of pre-existing patterns, sometimes resulting in novel, hybrid patterns due to epistatic effects; and 3) unidentified modifier genes can modulate the color output of Optix-positive scale cell precursors. Similar principles may apply to Optix-independent patterns.

## Conclusions

Multiple lines of evidence suggest that the regulatory evolution of the *optix* locus drives morphological radiations in the wings of *H. melpomene* and *H. erato*[15,18,30,45]. Regulatory evolution of this locus may also have a direct influence on species richness itself due to assortative mating between butterflies displaying identical red wing patterns [35,36]. In the light of the present data, we infer that the evolution of Optix color patterning roles sparked the diversification of red wing patterns within the Heliconiini tribe, notably among the spectacular mimetic radiations observed in the genera *Heliconius* and *Eueides*. Multiple recruitments of Optix expression into the differentiation of derived wing scales - the red color scale cells, as well as the male-specific vein structures of basal heliconiines - provide compelling examples of developmental innovation, and the genetic co-option event associated to coloration was followed by a rapid radiation in wing patterns. By opening paths to unexplored valleys of the morphospace, gene co-option events may be a general mechanism explaining sudden bursts of phenotypic change.

## Competing interests

The authors declare that they have no competing interests.

## Authors’ contributions

AM carried out wing development work, analyzed the data and drafted the manuscript. KJM carried out eye and brain immunostainings. NHP supervised antibody production. ADB provided live *Heliconius* pupae and carried out the spectrophotometer measurements. LEG performed the hybrid *Heliconius* crosses and contributed live *Heliconius* pupae. RDR supervised the project and helped to draft the manuscript. All authors read and approved the final manuscript.

## Supplementary Material

Additional file 1 **Martin_et_al-Supplementary.pdf.** Supplementary Materials including Supplementary Methods, Supplementary Table, Supplementary Figures 1– 6 and Supplementary References. Click here for file
