# Supplementary material for: Multiple recent co-options of Optix associated with novel traits in adaptive butterfly wing radiations
Source: EvoDevo. 2014 Feb 5;5:7. doi: 10.1186/2041-9139-5-7 (PMC3922110; doi:10.1186/2041-9139-5-7)
Supplement: Additional file 1 — Martin_et_al-Supplementary.pdf. Supplementary Materials including Supplementary Methods, Supplementary Table, Supplementary Figures 1– 6 and Supplementary References. [file 2041-9139-5-7-S1.pdf]

Additional File 1: Supplementary Material

Multiple recent co-options of Optix associated with novel traits in adaptive butterfly wing radiations

Arnaud Martin[1,2*], Kyle J. McCulloch[2], Nipam H. Patel[3], Adriana D. Briscoe[2], Lawrence E. Gilbert[4], Robert D. Reed[1]

[1]: Department of Ecology and Evolutionary Biology, Cornell University, Ithaca, NY 14853, USA.

[2]: Department of Ecology and Evolutionary Biology, University of California, Irvine, CA 92697, USA.

[3]: Department of Molecular and Cell Biology, University of California, Berkeley, CA 94720-3140, USA.

[4]: Department of Integrative Biology, University of Texas, Austin, Texas 78712, USA

*: to whom correspondence should be addressed; E-mail: heliconiuswing@gmail.com

1. Supplementary Methods

(a) Scanning electronic microscopy, *in situ* hybridizations and spectrophotometry

Scanning electron micrographs, *optix* mRNA detection by *in situ* hybridization in pupal wings, and reflectance measurements of adult wing scales were performed as previously described (1,2).

(b) Western Blotting

Two forewings were dissected during pupal development, at a stage where eye ommatidia were fully differentiated but showed no sign of pigmentation (pre-ommochrome stage). Each wing was lysed with a pestle in Laemmli buffer supplemented with 4ug of DNAseI, boiled for 5', and loaded on a 10% SDS-PAGE gel. Separated proteins were then transferred to a nitrocellulose membrane. Western blotting was performed using the anti-Optix polyclonal serum (dilution 1:10,000), an anti-rat IgG-HRP-linked secondary antibody (Cell Signaling Technology; dilution 1:5,000), and chemoluminescent detection on photosensitive film.

(c) Consensus phylogeny and ancestral character reconstruction

A concatenated consensus phylogeny was constructed by concatenation of previously published molecular phylogenies (Supplementary Figure 1). First, we applied a stringent cut-off on node support to convert to polytomies the unresolved nodes of five phylogenetic trees spanning various depth of the Heliconiinae phylogeny and using different molecular character sets (3–6). Then, the CONSENSE module of the PHYLIP package was used to build a consensus topology following the extended Majority Rule (*mre*) method (7). As our input trees showed no conflicts between resolved nodes, this consensus tree method is equivalent to giving priority to resolved nodes over polytomies: for instance, the concatenation of [a, b, c, d], [(a, b), c, d] and [a, b, (c, d)] results in the [(a, b), (c, d)] topology. We then used this consensus phylogeny to perform ancestral character state reconstruction using the two-parameter likelihood method implemented in

Mesquite (8). For scoring the presence/absence of male-specific vein scales (Fig. 4), we examined a minimum of two specimens per sex and species under a binocular microscope. Specimens used for character mapping originated from a private collection of Dr. Adriana Briscoe (Heliconiini) and from the Cornell University Insect Collection (non-Heliconiini outgroups). For scoring the "FW short bar" and "HW base" characters (Supplementary Figure 5), and extended the phylogenetic sampling by examining online photographs of each species present on the consensus tree (Supplementary Figure 4). For each American species, all the photographs featured on the Butterflies of America website (http://www.butterfliesofamerica.com; accessed in May 2013) as well as butterflies originating from the collection of Dr. Briscoe (N>3 per species) were scrutinized. For non-American species, we inspected a minimum of five high-quality photographs per species originating from various online sources. The "FW short bar" was coded as present after observation of a ventral-specific short pink/red/brown/orange bar at the base of the forewing costa. The "HW base" character corresponded to one or several ventral specific small pink/red/brown/orange spots at the base of the hindwing tracheal trunk.

2. Supplementary Table

Supplementary Table. Specimens used for the immunodetection of Optix in pupal wings

**Species/morph**	**Source** (*: natural population)	**Time of dissection day of pupation = day1**	**Nb of individuals**	**Phenotypic variablity in stock**
Dryas iulia	Costa Rica Entomological Supplies	day of arrival	3	Sexual dimorphism on dorsal forewing veins
Heliconius charitonia vasquezae	Costa Rica Entomological Supplies	day of arrival	2	none
Heliconius doris	Costa Rica Entomological Supplies	day of arrival	10	out of 33 adults that were let emerged: 26 red morphs, 4 blue morphs, 3 green morphs
Heliconius erato petiverana	Costa Rica Entomological Supplies	day of arrival	8	none
Heliconius melpomene rosina	Costa Rica Entomological Supplies	day of arrival	9	residual hybridism with *H. cydno* ; 2 putative hybrids were ruled out
Heliconius sapho leuce	Costa Rica Entomological Supplies	day of arrival	4	none
Heliconius sara sara	Costa Rica Entomological Supplies	day of arrival	2	none
Heliconius antiochus	Greenhouse colony at University of Texas - Austin	day4-5 after pupation	3	none
Heliconius atthis	Greenhouse colony at University of Texas - Austin	day5 after pupation	9	none
Heliconius hecale fornarina	Greenhouse colony at University of Texas - Austin	day5 after pupation	3	Variable shape and size of the ventral hindwing black patterns, as in natural populations
Heliconius melpomene - forewing "narrow red" hybrid population	Greenhouse colony at University of Texas - Austin	day4-5 after pupation	4	Stable position and shape of forewing red/white boundary
Euphydryas chalcedona chalcedona	*Keckiella cordifolia* plants in Azusa, CA*	day4-5 after pupation	5	Continous variation on the size and relative composition of Dorsal Orange + Black patterns
Nymphalis antiopa	Pre-pupae found around Steinhaus building in mid-April, UC Irvine, CA*	day4-5 after pupation	8	none
Agraulis vanillae	*Passiflora caerulea* plants in Huntington Beach, CA*	day4-5 after pupation	6	Sexual dimorphism on dorsal forewing veins

3. Supplementary Figures

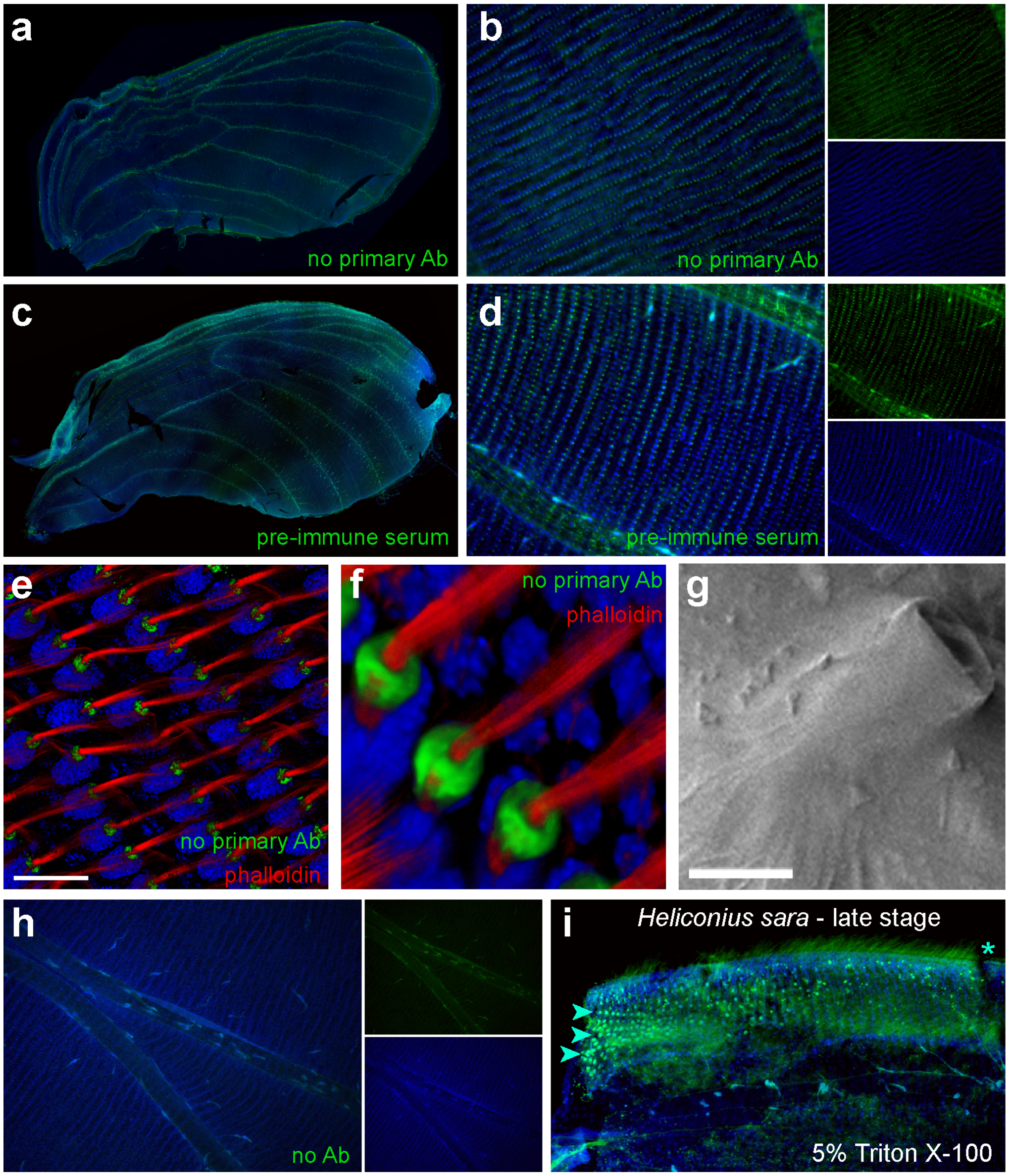

Supplementary Figure 1. Negative controls for artifactual signals during Optix immunodetection. (a-b) Veins and scale socket background signal observed in a *H. erato petiverana* forewing incubated with an anti-rat IgG secondary antibody only. **(c-d)** A similar background is observed at identical exposure times when the anti-Optix serum is replaced by the pre-immune serum originating from the same donor animal (dilution 1:3000). **(e-f)** Scale socket background signals observed in negative control incubations. Phalloidin was used to visualize protruding F-actin filaments of the developing scales. Scale bar: 20µm **(g)** Scanning electron micrograph of an adult scale socket. Scale bar: 5µm. **(h)** Vein auto-fluorescence background in a *H. erato petiverana* forewing photographed as in panels (b,d). **(i)** Dissected tissues showing extensive scale emergence on the wing margin are impermeable to antibody penetration. Incomplete immune-detection of Optix is shown here (arrowheads) upon a ten-fold increase in detergent concentration, in the ventral forewing bar of a *Heliconius sara* specimen dissected after scale emergence (asterisk: background signal in wing margin scales).

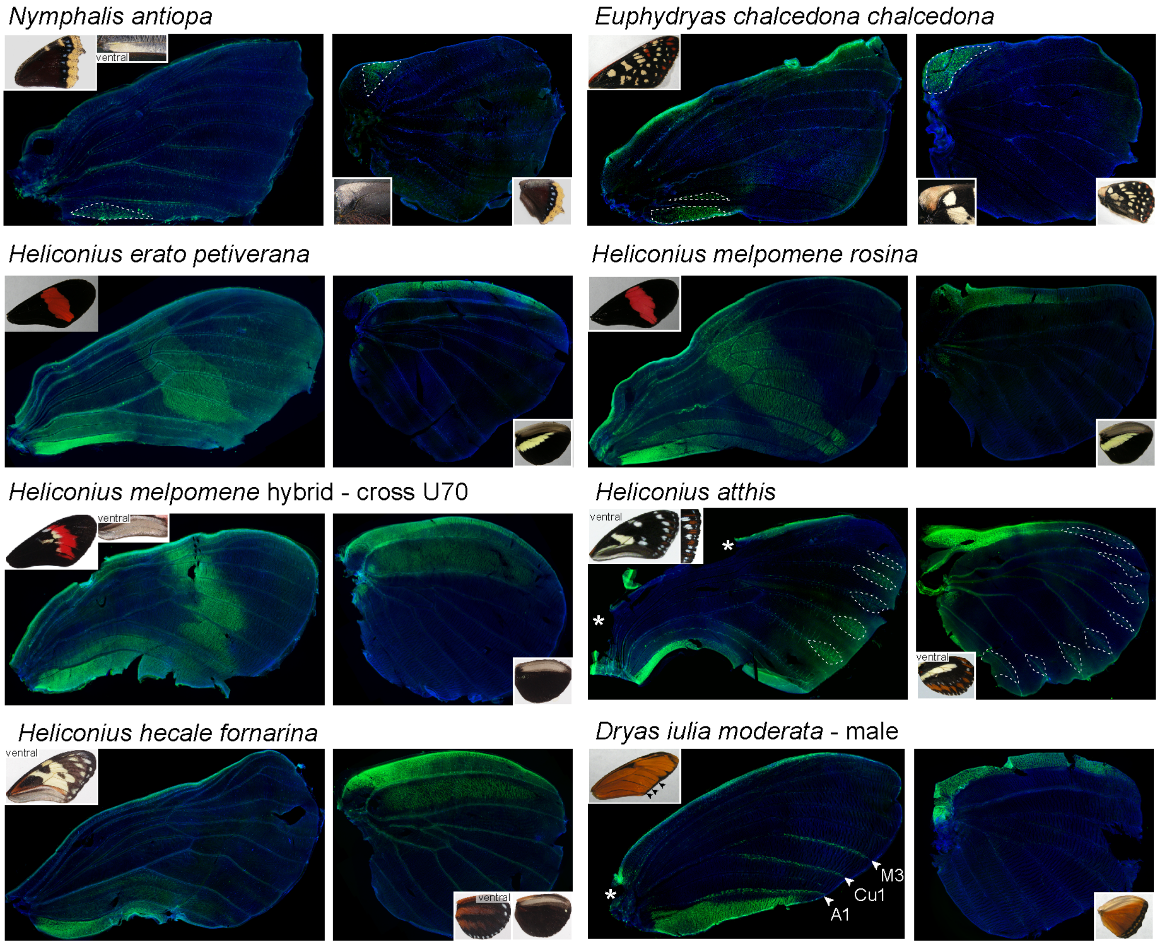

Supplementary Figure 2. Comparative immunodetection of Optix during pupal development. In addition to conserved expression domains identified in all butterflies assessed (anterior forewing blade; forewing ventro-posterior and hindwing dorso-anterior patches of acute scales), Optix marks Heliconinii red patterns as well as forewing-specific vein-scale in *Dryas iulia* males. Insets features dorsal views of adult wings, unless otherwise noted. Dotted lines: fields of Optix-positive scale cell precursors ; asterisks: missing Optix signals due to local tissue damage.

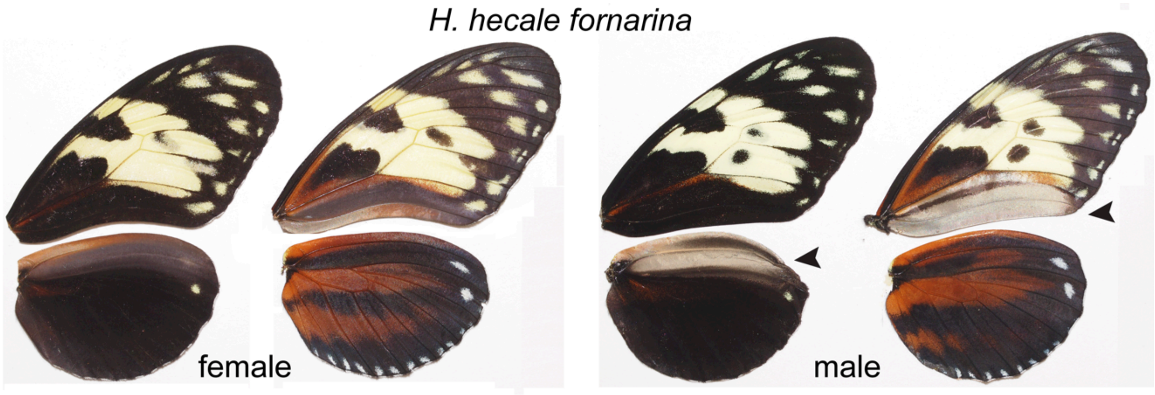

Supplementary Figure 3. Sexual dimorphism in patches of wing coupling scales. Females of *H. hecale fornarina* display narrow patches of wing coupling scales on the posterior edge of the ventral forewing and on the anterior edge of the dorsal hindwing. Males show a dramatic expansion of these surfaces (arrowheads, grey scales). This male-specific expansion is not a rule but is nonetheless widespread across *Heliconius*, for instance in the *H. melpomene* clade and in *H. sapho* where similar sex differences are conspicuous. We suggest that differences in frictional surface between wings may optimize sex-specific flight dynamics such as foraging, mate location strategy and courtship (9).

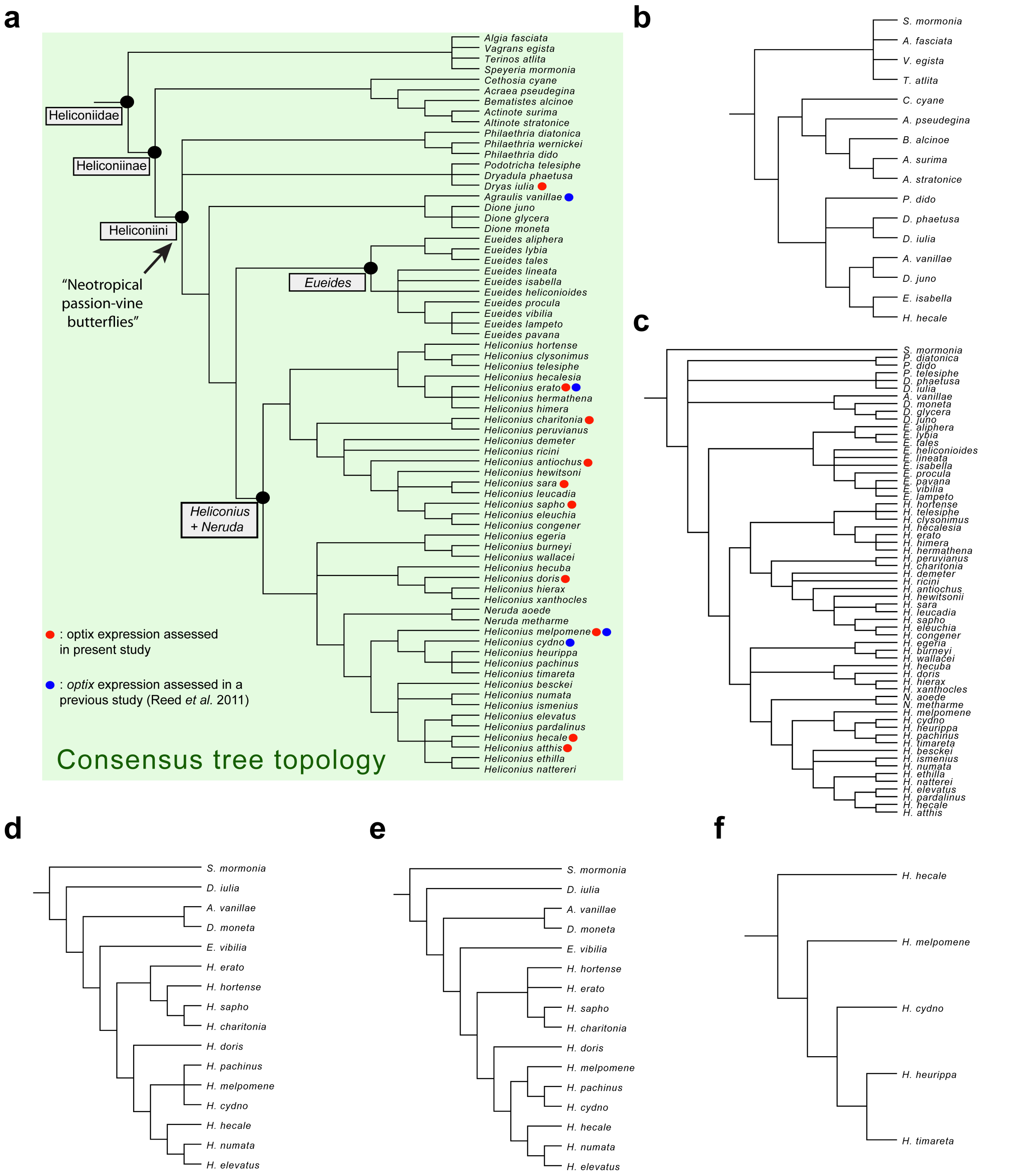

Supplementary Figure 4. Consensus phylogeny of Heliconiini used for character mapping. (a) Consensus tree topology obtained by concatenation of consensus trees derived from previous molecular phylogenies by using a priority rule on resolved dichotomies. The tree contains all the known Heliconiini genera and features 61 species out of 70 assigned to this tribe (10). **(b-f)** Source tree topologies used to build the concatenated consensus topology; in all cases, nodes with bootstrap support <80% were removed to generate polytomies that were eventually resolved by a complementary study. **(b)** Sub-tree derived from a multigenic molecular phylogeny of Nymphalidae (4). **(c)** Tree derived from a multigenic molecular phylogeny of Heliconiini (3). **(d-e)** Tree derived from gene phylogenies of the *BRh* **(d)** and *LWRh* **(e)** genes across Heliconiini (5). **(f)** Tree derived from a genome-wide phylogenetic analysis of the *H. melpomene-cydno* clade (6).

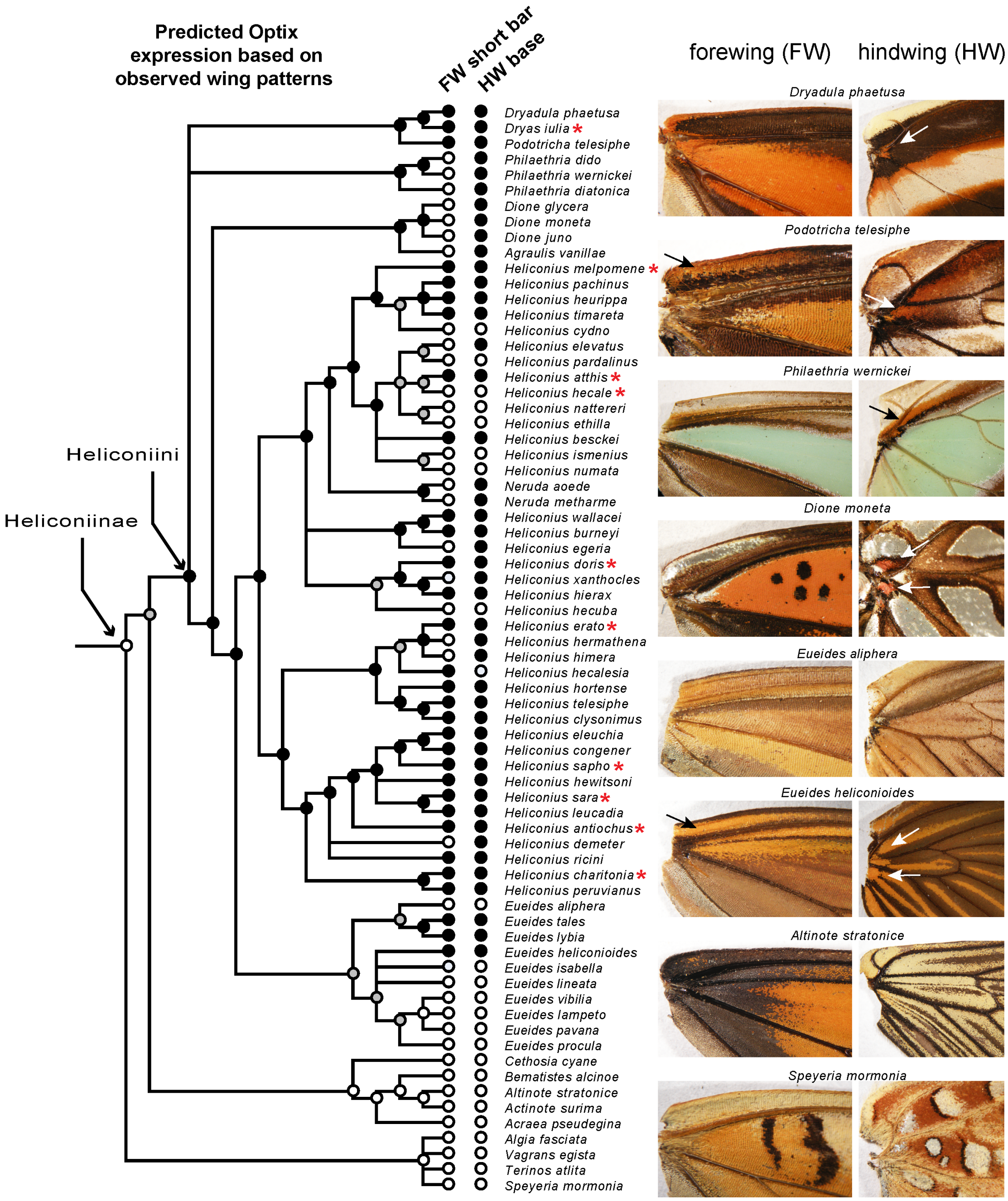

Supplementary Figure 5. Optix-positive wing patterns of the proximal complex are a synapomorphy of Heliconiini. Character mapping of the red ventral forewing short bar and red ventral hindwing basal spots (arrows) characteristic of Optix expression in Heliconiini (black: predicted presence ; white: predicted absence); asterisk: state defined by Optix immunodetection (Figure 2). Ancestral character state reconstruction is shown here at internal nodes for the ventral hindwing spots (grey: equivocal state), with a probability of character loss = 20x probability of gain. Character states were assigned by counting ambiguous cases as negative (*e.g.* silver forewing bar of *Dione spp.*; see Supplementary Methods). Most importantly, the basal patterns characteristic of Optix expression (ventral short bar and well defined red basal spots) are not observed outside of Heliconiini on large collections of butterfly specimens (*e.g.* http://www.butterfliesofamerica.com), suggesting that the color patterning role of Optix is a synapomorphy of Heliconiini.

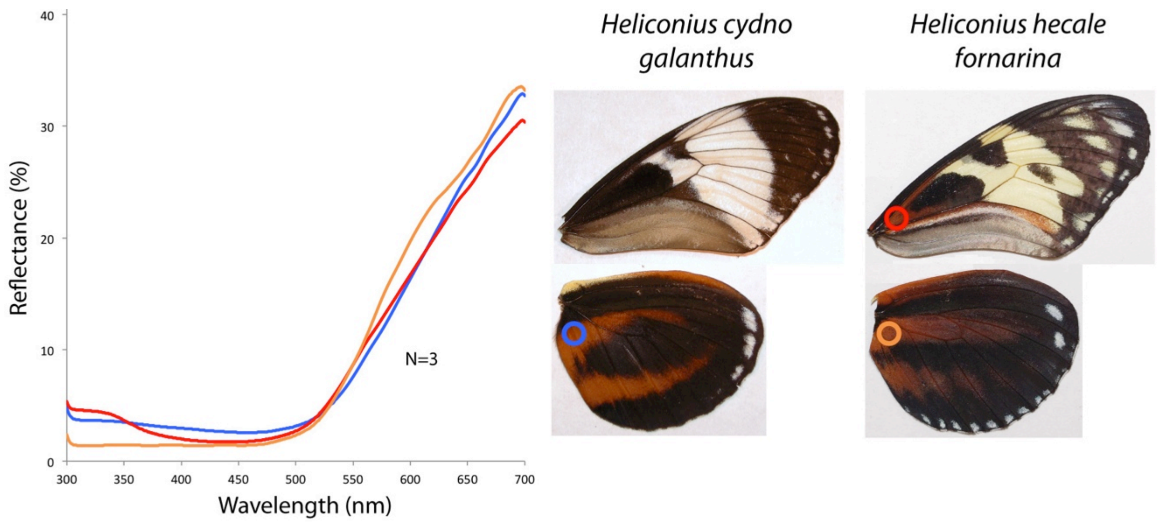

Supplementary Figure 6. Spectral resemblance between the ventral red colors of sympatric butterflies. Spectrophotometric measurement of light reflectance of the butterflies *H. cydno galanthus* and *H. hecale fornarina* reveals comparable color compositions between the ventral red patterns (bullets) of these co-occurring morphs (Guatemala, El Salvador). Each line is averaged from three measurements. See Ref. 2 (Supplementary Online Material: figure 2) for spectrophotometer measurements of a wide variety of color patterns in Heliconiini.

4. Supplementary References

1. Reed RD, Papa R, Martin A, Hines HM, Counterman BA, Pardo-Diaz C, et al. optix drives the repeated convergent evolution of butterfly wing pattern mimicry. Science. 2011 Aug 26;333(6046):1137–41.

2. Briscoe AD, Bybee SM, Bernard GD, Yuan F, Sison-Mangus MP, Reed RD, et al. Positive selection of a duplicated UV-sensitive visual pigment coincides with wing pigment evolution in Heliconius butterflies. Proc Natl Acad Sci. 2010 Feb 23;107(8):3628–33.

3. Beltran M, Jiggins CD, Brower AVZ, Bermingham E, Mallet J. Do pollen feeding, pupal-mating and larval gregariousness have a single origin in Heliconius butterflies? Inferences from multilocus DNA sequence data. Biol J Linn Soc. 2007 Oct 1;92(2):221–39.

4. Wahlberg N, Leneveu J, Kodandaramaiah U, Peña C, Nylin S, Freitas AVL, et al. Nymphalid butterflies diversify following near demise at the Cretaceous/Tertiary boundary. Proc R Soc B Biol Sci. 2009 Dec 22;276(1677):4295–302.

5. Yuan F, Bernard GD, Le J, Briscoe AD. Contrasting Modes of Evolution of the Visual Pigments in Heliconius Butterflies. Mol Biol Evol. 2010 Oct 1;27(10):2392–405.

6. Nadeau NJ, Martin SH, Kozak KM, Salazar C, Dasmahapatra KK, Davey JW, et al. Genome-wide patterns of divergence and gene flow across a butterfly radiation. Mol Ecol. 2013 Feb 1;22(3):814–26.

7. Felsenstein, J. PHYLIP (Phylogeny Inference Package) version 3.5c. Distributed by the author. Department of Genetics, University of Washington, Seattle; 1993.

8. Madison WP, Madison DR. Mesquite: a modular system for evolutionary analysis [Internet]. 2007. Available from: http://mesquiteproject.org

9. Mendoza-Cuenca L, Macías-Ordóñez R. Foraging polymorphism in Heliconius charitonia (Lepidoptera: Nymphalidae): morphological constraints and behavioural compensation. J Trop Ecol. 2005;21(04):407–15.

10. Lamas G, Callaghan C, Casagrande MM, Mielke O, Pyrcz T, Robbins R, et al. Hesperioidea - Papilionoidea. In: Heppner JB, editor. Atlas Neotropical Lepidoptera Checkl Part 4A. Gainesville, Florida: Association for Tropical Lepidoptera/Scientific Publishers; 2004.
